# Supplementary material for: Probing phonon transport dynamics across an interface by electron microscopy
Source: Nature. 2025 Jun 11;642(8069):941–6. doi: 10.1038/s41586-025-09108-6 (PMC12738285; doi:10.1038/s41586-025-09108-6)
Supplement: Supplementary file 1 — Supplementary Information [file 41586_2025_9108_MOESM1_ESM.pdf]

nature portfolio

https://doi.org/10.1038/s41586-025-09108-6

Supplementary information

Probing phonon transport dynamics across an interface by electron microscopy

In the format provided by the authors and unedited

Supplementary Information for Probing phonon transport dynamics across an interface by electron microscopy

Fachen Liu[1,2,7], Ruilin Mao[1,7], Zhiqiang Liu[6], Jinlong Du[1], Peng Gao[1,2,3,4,5] ✉

[1] International Center for Quantum Materials, and Electron Microscopy Laboratory, School of Physics, Peking University, Beijing, China.

[2] Academy for Advanced Interdisciplinary Studies, Peking University, Beijing, China.

[3] Tsientang Institute for Advanced Study, Zhejiang, China

[4] Interdisciplinary Institute of Light-Element Quantum Materials and Research Center for Light-Element Advanced Materials, Peking University, Beijing, China.

[5] Collaborative Innovation Center of Quantum Matter, Beijing, China.

[6] Research and Development Center for Solid State Lighting, Institute of Semiconductors, Chinese Academy of Science, Beijing, China.

[7] These authors contributed equally to this work

✉ e-mail: pgao@pku.edu.cn

Supplementary Text 1: Probability of three-phonon scattering process

Here is a supplementary explanation regarding the relationship between the scattering probability and the population of each mode for a specific three-phonon system which has already satisfied the selection rule. The probability of phonon scattering with other phonons depends on population of the initial and final states [1,2]. Consider three phonon modes λ_1, λ_2 and λ_3 whose energy satisfy $\omega_1 + \omega_2 = \omega_3$, and momentum satisfy $\boldsymbol{q_1} + \boldsymbol{q_2} + \boldsymbol{q_3} = \boldsymbol{G}$. Using the particle number representation, the present state can be expressed as $|n_1, n_2, n_3\rangle$. Since the three-phonon scattering process is reversible, the system can undergo the absorption process $P^+(\lambda_1 + \lambda_2 \rightarrow \lambda_3)$ to the state $|n_1 - 1, n_2 - 1, n_3 + 1\rangle$, or the emission process $P^-(\lambda_3 \rightarrow \lambda_1 + \lambda_2)$ to the state $|n_1 + 1, n_2 + 1, n_3 - 1\rangle$. According to the relationship between scattering probability and initial and final state [3]

$$P^+ = \frac{\pi}{4\rho^3 N_0 \Omega} \frac{q_1 q_2 q_3}{c_1 c_2 c_3} |A_{123}|^2 n_1 n_2 (n_3 + 1) \times \delta_{\boldsymbol{q_1}+\boldsymbol{q_2}+\boldsymbol{q_3},\boldsymbol{G}} \delta(\omega_1 + \omega_2 - \omega_3) \quad (1A)$$

$$P^- = \frac{\pi}{4\rho^3 N_0 \Omega} \frac{q_1 q_2 q_3}{c_1 c_2 c_3} |A_{123}|^2 (n_1 + 1)(n_2 + 1) n_3 \times \delta_{\boldsymbol{q_1}+\boldsymbol{q_2}+\boldsymbol{q_3},\boldsymbol{G}} \delta(\omega_3 - \omega_1 - \omega_2) \quad (1B)$$

Symmetry guarantees that $\frac{\pi}{4\rho^3 N_0 \Omega} \frac{q_1 q_2 q_3}{c_1 c_2 c_3} |A_{123}|^2$ is perfectly symmetrical to the three phonons, unchanged by exchange.

Now we define the forward process P_{forward} as the net absorption process,

$$P_{\text{forward}} = P^+ - P^- = \frac{\pi}{4\rho^3 N_0 \Omega} \frac{q_1 q_2 q_3}{c_1 c_2 c_3} |A_{123}|^2 [n_1 n_2 - n_3(n_1 + n_2 + 1)] \quad (2)$$

The initial states of P_{forward} are λ_1 and λ_2. The partial derivation of the initial state population n_1 is

$$\frac{\partial}{\partial n_1} P_{\text{forward}} = \frac{\pi}{4\rho^3 N_0 \Omega} \frac{q_1 q_2 q_3}{c_1 c_2 c_3} |A_{123}|^2 (n_2 - n_3) \quad (3)$$

Thus, as long as $n_2 > n_3$ (always satisfied unless the population is inversed, i.e., the population of the high-energy state exceeds that of the low-energy state), $\frac{\partial}{\partial n_1} P_{\text{forward}}$ will always be positive, meaning that the net absorption process P_{forward} will be more likely to occur as the initial state n_1 increases. The net process of emission process is $P_{\text{reverse}} = P^- - P^+ = -P_{\text{forward}}$ with λ_1 and λ_2 as the final states and λ_3 as the initial state. Obviously, $\frac{\partial}{\partial n_1} P_{\text{reverse}}$ is negative (as long as $n_2 > n_3$), which means that the net emission process will be more likely to occur as the final state n_1 decreases. Now take the partial derivative of n_3 with respect to the P_{reverse}

$$\frac{\partial}{\partial n_3} P_{\mathrm{reverse}} = \frac{\pi}{4\rho^3 N_0 \Omega} \frac{q_1 q_2 q_3}{c_1 c_2 c_3} |A_{123}|^2 (n_1 + n_2 + 1) \tag{4}$$

Obviously, $\frac{\partial}{\partial n_3} P_{\mathrm{reverse}}$ is always positive and $\frac{\partial}{\partial n_3} P_{\mathrm{forward}}$ is always negative, which conforms to the law that the net process (whether absorption or emission) is more likely to occur as the initial state population increases and the final state population decreases.

Supplementary Text 2: Discussion of the concept of detailed balancing and feasibility of using EELS to measure temperature under temperature gradients

The "detailed balance" introduced in this work is the principle of detailed balance for high-energy electron-phonon interactions. One of the fundamental approximations of electron-phonon interaction is the "frozen lattice" approximation. One of the most important elements of this hypothesis is that the specimen thickness and the mean-free-path length for phonon excitation are both smaller than the distance travelled by the electron within the lifetime of the phonon [4]. That is, the time for the electron-phonon scattering process to establish equilibrium is much shorter than the average lifetime of the phonon. Thus, what the electron actually "sees" is the population of phonon in a non-equilibrium state. In previous experimental studies, some researchers have also directly introduced nonequilibrium phonon population number into the scattering cross-section formula [5].

Based on the above approximation, we introduce a non-equilibrium population number formulation under temperature gradients [6], as shown in Equation (5):

$$f_{\sigma,k} = f_0\big(\omega_\sigma(k)\big) - \tau v_{\sigma,i}(k)\frac{\partial f_0}{\partial T}\frac{\partial T}{\partial x_i} \tag{5}$$

where f_0 is the Bose-Einstein distribution function at equilibrium, τ is the relaxation time of the phonon, and v is the phonon group velocity at the corresponding momentum point.

Considering the processes of electron and phonon scattering in materials, the principle of detailed balancing rule requires:

$$\frac{P_{loss}}{P_{gain}} = \frac{I_{loss}}{I_{gain}} = \frac{\langle n\rangle + 1}{\langle n\rangle} \tag{6}$$

where $\langle n\rangle$ is the Bosonic distribution under equilibrium states. Then the ratio of the electron energy loss to the electron energy gain spectral intensity in the equilibrium state satisfies:

$$\frac{I_{loss}}{I_{gain}} = \exp\left(\frac{\hbar\omega}{k_B T}\right) \tag{7}$$

Substituting Equation (5) into Equation (6), we obtain Equation (8):

$$\frac{I_{loss}}{I_{gain}} = \frac{\beta - \sigma}{\frac{\beta}{\beta + 1} - \sigma} \tag{8}$$

where $\beta = \exp\left(\frac{\hbar\omega}{k_B T}\right) - 1, \sigma = \tau v_i \frac{\partial T}{\partial x_i}\frac{\hbar\omega}{k_B T^2}$. While $\frac{\partial T}{\partial x_i} = 0, \sigma = 0$, the system is at equilibrium state, then the equation degenerates to Equation (7).

We have solved this model exactly numerically. For wurtzite-AlN optical phonons, the mean free path of TO phonons at 300 K is approximately 1 nm [7], and the mean free path can be approximated as the product of the phonon relaxation time τ and the phonon group velocity *v*. The typical temperature gradient in the non-interface regions of the heated sample is 0.18 K/nm. We selected temperature gradients of 0 K/nm, 0.18 K/nm (temperature gradient in bulk measured by our experiments), 5 K/nm, 20 K/nm, and 60 K/nm to plot the relationship between $\log(I_{loss}/I_{gain})$ and ω, as shown in Figure S6. It can be seen from Figure S6 that the non-equilibrium $\log(I_{loss}/I_{gain})$ curves still pass through the origin, but produce a nonlinear trend and a slope change than the equilibrium curve at high energy region. Within 5K /nm (orange line), the temperature gradient only slightly affects the slope of the curve, which is basically within the experimental error range.

In this work, we measured the temperature map at the micrometer scale, and all the measured temperature gradients in bulk did not exceed 0.18 K/nm (red line), which is almost no difference from the temperature fitting in the equilibrium state, indicating the applicability of the local near equilibrium approximation. While the temperature drop near the interface occurs in the range of ~2nm, and the degree of non-equilibrium is two to three orders of magnitude higher than in bulk, enough to cause significant non-equilibrium effects. Considering the complexity of defining temperature under high non-equilibrium degree at interface, we do not focus on the exact value of temperature in the related discussion in Figure 3.

Supplementary Text 3: Discussion of the uncertainties in temperature measurement

The discussion in the main text addresses two types of uncertainty. The first (shown in Fig. 2b) is the uncertainty of the temperature calculation at a single data point, defined as $\pm 3\sigma$ (99.7% confidence interval) of the least squares fit result of temperature. The second uncertainty, represented by the shaded region in Fig. 2e, is the uncertainty of the average temperature obtained from multiple data points (equivalent to the error bar), derived from the standard deviation between the results of multiple acquisitions at the same position.

In our study, the uncertainty of the temperature measurement (Figures 2b, 2c, and 2e) mainly come from the quantitative ratio of EEL and EEG signals. In this case, the signal-to-noise ratio of EEG is the key to determine the uncertainty as the EEL signal is much stronger. In order to improve the signal-to-noise ratio of EEG, we have optimized experimental conditions. Firstly, the experiments were performed at relatively higher temperature. In this case, the EEG signals are stronger based-on the Bose Einstein relation. Secondly, in order to achieve high counts of EEG signal, it is certainly beneficial to increase the integration time of the spectra acquisition. However, on the other hand, the aberration and current of electron-beam changes over time, leading to degradation of the resolution and stability of the electron-probe, which will make an additional contribution to the second type of uncertainty. So, optimization of the acquisition time is important, and it is beneficial to adopt a longer integration time under the premise that the ZLP shape is almost unchanged. In addition, if multiple acquisition and superposition can be carried out as other conditions remain unchanged, the second type of uncertainty will be reduced. Thirdly, although the higher energy resolution is better for background removal, the highest energy resolution can be only achieved at very low electron beam current, which corresponds to low signal-to-noise ratio of spectra. Again, we have optimized the energy resolution and electron counts by adjusting the EELS parameters to obtain a high energy resolution while maintaining a relatively large beam current.

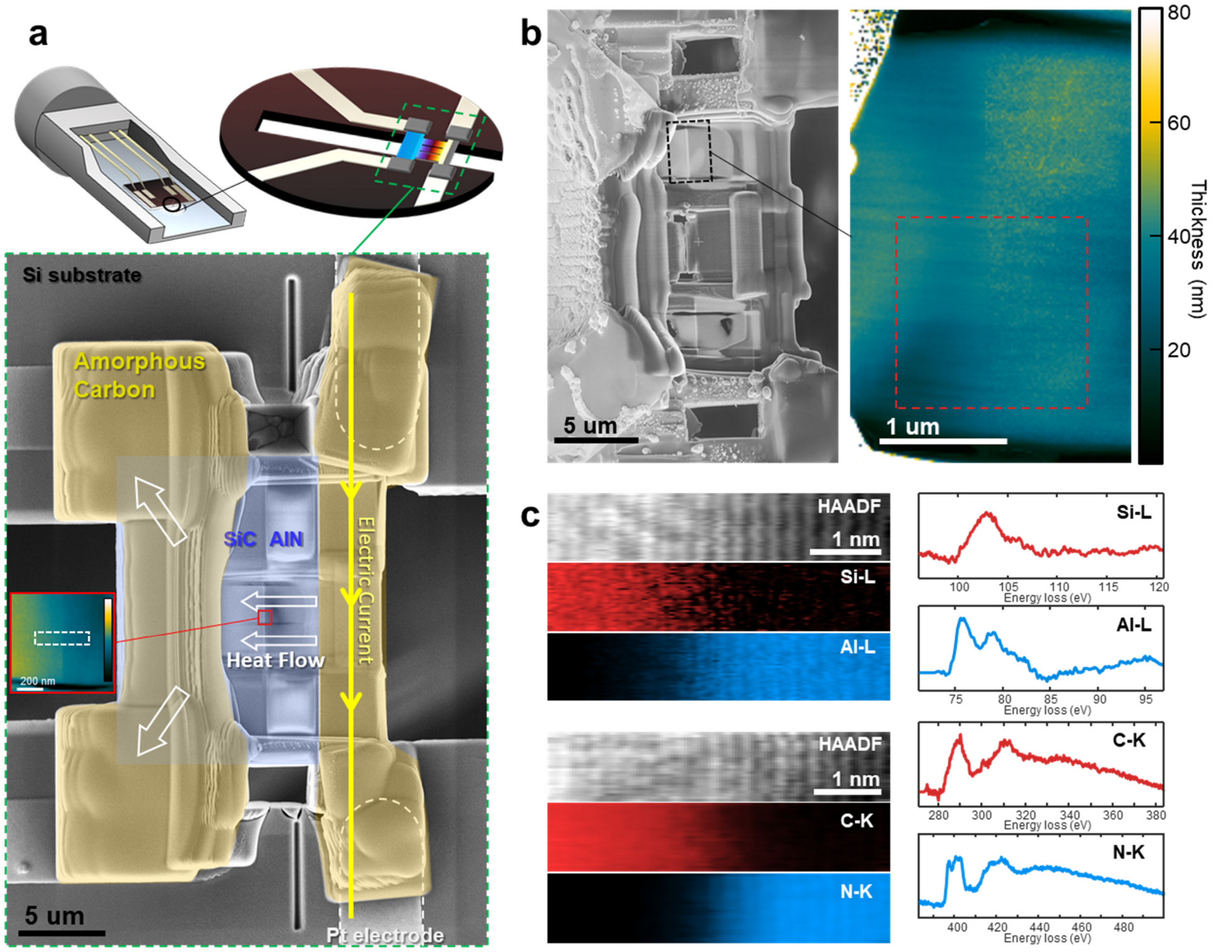

Figure. S1 | Preparation method and structure characteristics of the sample. a, Schematic diagram and scanning electron microscope image showing the sample prepared by focused ion beam for in situ STEM-EELS experiment. Amorphous carbon (yellow shades) was deposited on both sides of the sample (blue shade). The right one is used for Joule heating and the left one is used to connect the silicon substrate as the heat sink. The illustration shows the thickness (color bar 0-60 nm) of the interface area marked by the red box, and the white dashed box marked the collection area of Fig. 2e, whose thickness is 25~35 nm. **b,** Thickness mapping from EELS showing the typical thickness of the acquisition area in Fig. 2c is about 30~40 nm. Two sides of the foil sample are cut through to satisfy the one-dimensional heat transfer model. The red dashed box marked the collection area of Fig. 2c. **c,** Core-loss EEL signal showing the elemental distribution near the interface. Typically, the EELS measured intermixing width is less than 1 nm.

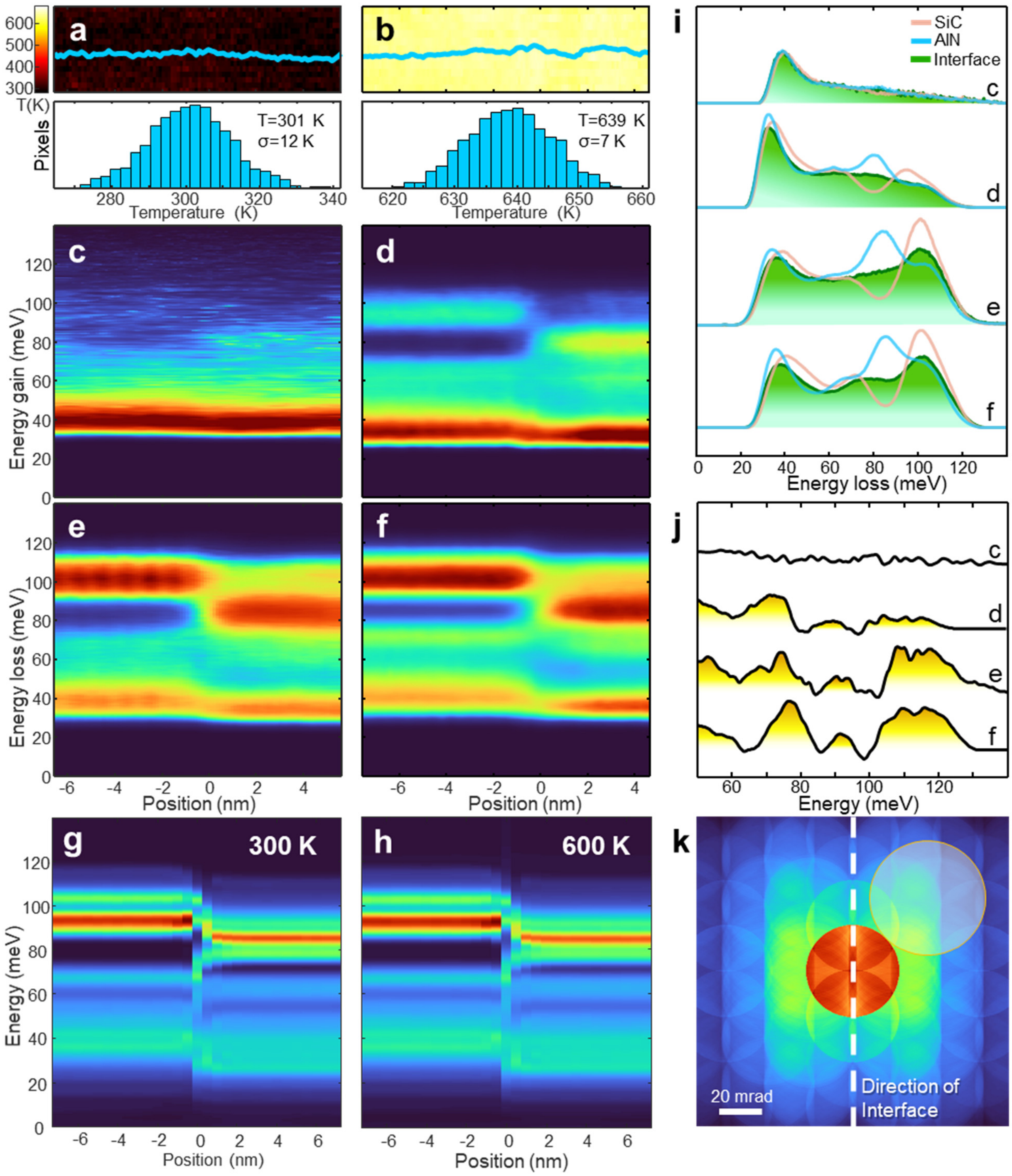

Figure. S2 | Spectral characteristics, calculation results of equilibrium states and off-axis direction used in experiments. a, b, Experimental fitted temperature map at room temperature without heating (a) and on a uniformly heated sample at ~640 K without temperature gradients (b). **c, d,** EEG spectra across the interface at room temperature (c) and high temperature (d). **e, f,**

Corresponding EEL spectra. **g, h,** Projected phonon DOS near the interface at 300 K (g) and 600 K (h) calculated from molecular dynamics, showing no significant difference between 300K and 600K except for a slight redshift (<2 meV). **i,** Phonon spectra extracted from (c-f) at the position of SiC (red line), AlN (blue line) and interface (green shade). **j,** Interface residual spectra after removing the spectral components of bulk SiC and AlN using the least squared fitting method. At room temperature, EEG signal of curve C is too weak to extract interface residual signal. In equilibrium at high temperature of curve D, both α and β modes have a certain intensity in EEG signal, which is consistent with EEL signal, indicating that the absence of non-equilibrium population distribution without heat flow. **k,** Relative direction between EELS entrance aperture and diffraction disk. The red circle and green circles are transmission disk and diffraction disks respectively. The yellow disk represents the EELS entrance aperture, with its center oriented at a 45° angle to the interface, which can keep most of modes along Γ-A direction have high excitation activity.

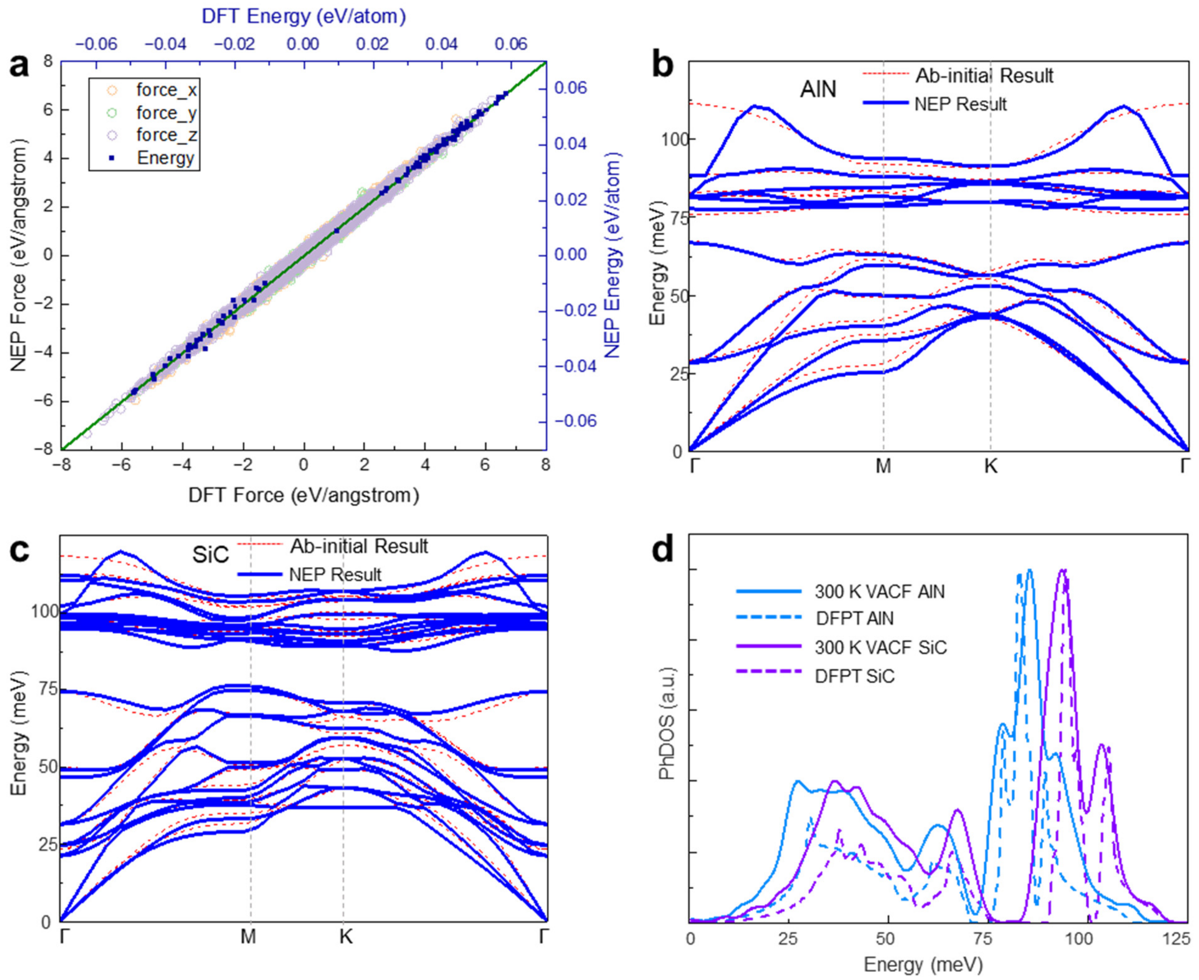

Figure. S3 | Validation of NEP Potentials. a, Atomic energies and forces obtained from AlN/SiC NEP and ab initio calculations for the testing dataset. **b, c,** Comparison of ab-initial phonon spectrum of AlN (b) and SiC (c). The LO-TO splitting effect was not considered while using NEP. **d,** Comparison of Phonon DOS calculated from velocity autocorrelation function (VACF) of 300 K MD trajectory and Density Functional perturbation theory (DFPT).

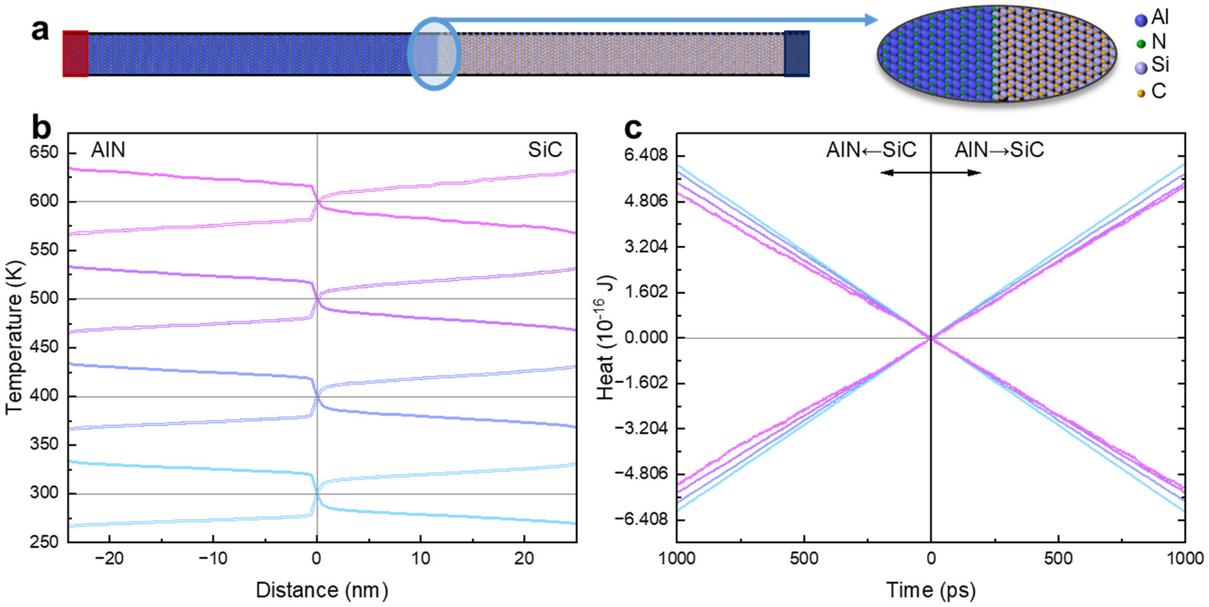

Figure. S4 | Details of NEMD Simulations. a, The interface structure used in NEMD simulation. The structure is a hexagonal lattice with an in-plane cell parameter of 3.75 nm and an out-plane cell parameter of 72.2 nm. The enlarged image on the right shows the atomic configuration near the interface, with termination surfaces of Si and N. **b,** Temperature line profiles of NEMD simulation at different temperatures and heat current directions. **c,** Accumulated heat of NEMD simulation at different temperatures and heat current directions.

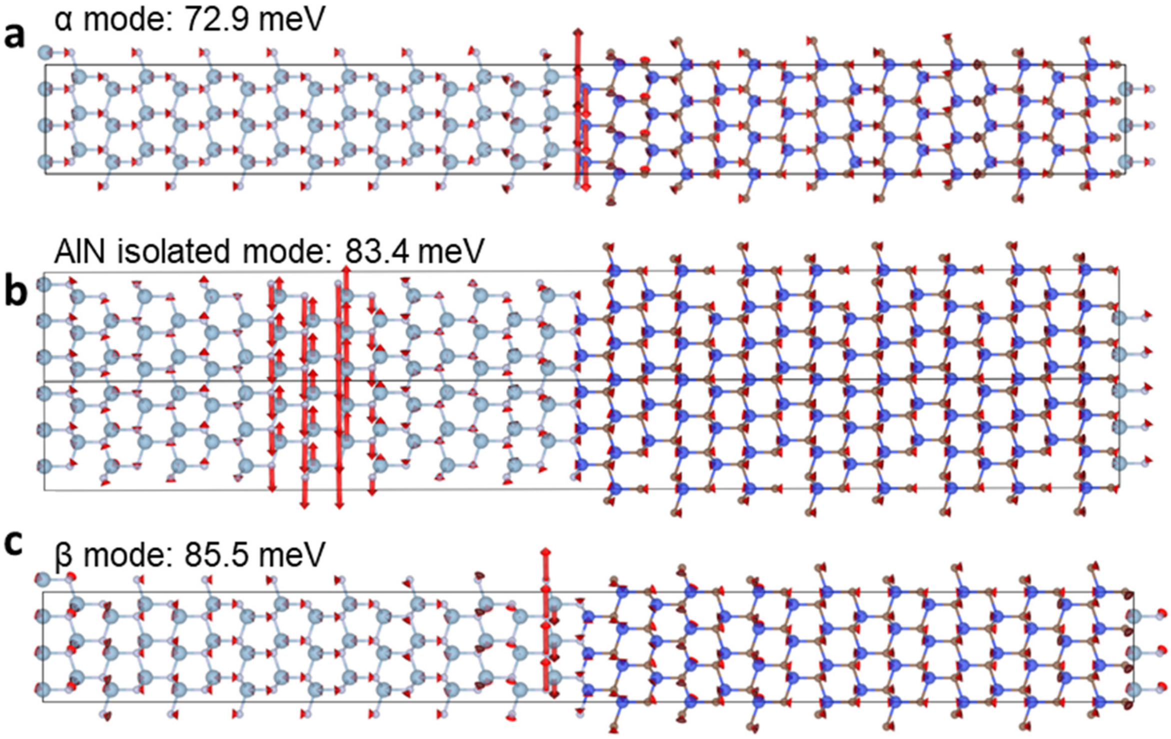

Figure. S5 | Vibrational eigenvectors of interfacial modes α and β (a, c) and an isolated mode in AlN (b). For clarity, the amplitudes of eigenvectors were magnified by a factor of five.

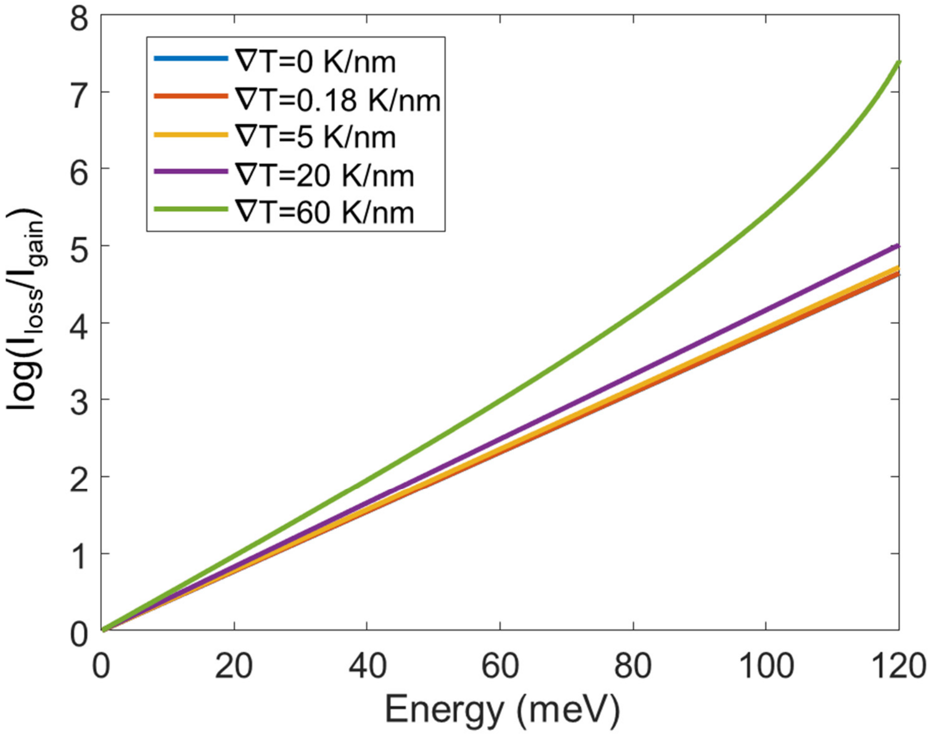

Figure S6 | Function of log(I_{loss}/I_{gain}) versus ω under temperature gradient induced non-equilibrium state. Blue line (∇T=0 K/nm) and orange line (∇T=0.18 K/nm) are too close to be distinguished.

Table S1

Calculated interfacial temperature drop, interfacial thermal resistance and thermal rectification ratio at different temperatures and heat flow directions.

	ΔT_+(K)	ΔT_-(K)	R_+(m^2K/GW)	R_-(m^2K/GW)	R_-/R_+
300K	36.51	36.40	0.70	0.72	1.0051
400K	32.67	33.06	0.68	0.72	1.0028
500K	29.36	30.83	0.63	0.68	1.0611
600K	23.47	25.50	0.54	0.61	1.1483

References

1. Li, W., Carrete, J., A. Katcho, N. & Mingo, N. ShengBTE: A solver of the Boltzmann transport equation for phonons. *Comput. Phys. Commun.* **185**, 1747–1758 (2014).
2. Ravichandran, N. K. & Broido, D. Phonon-Phonon Interactions in Strongly Bonded Solids: Selection Rules and Higher-Order Processes. *Phys. Rev. X* **10**, 021063 (2020).
3. Srivastava, G. P. *The Physics of Phonons*. (CRC Press, Abingdon, Oxon ; Boca Raton, FL, 2023).
4. Wang, Z. The Frozen-Lattice Approach for Incoherent Phonon Excitation in Electron Scattering. How Accurate Is It? *Acta Crystallogr. A* **54**, 460–467 (1998).
5. Gadre, C. A. *et al.* Nanoscale imaging of phonon dynamics by electron microscopy. *Nature* **606**, 292–297 (2022).
6. Hamada, M., Minamitani, E., Hirayama, M. & Murakami, S. Phonon Angular Momentum Induced by the Temperature Gradient. *Phys. Rev. Lett.* **121**, 175301 (2018).
7. AlShaikhi, A. & Srivastava, G. P. Phonon intrinsic mean free path in zincblende AlN. *Proc. Diam. 2006 17th Eur. Conf. Diam. Diam.- Mater. Carbon Nanotub. Nitrides Silicon Carbide* **16**, 1413–1416 (2007).
